# Tumor-Derived Autophagosomes (DRibbles) Induce B Cell Activation in a TLR2-MyD88 Dependent Manner

**DOI:** 10.1371/journal.pone.0053564

**Published:** 2013-01-09

**Authors:** Weixia Li, Meng Zhou, Hongyan Ren, Hong-Ming Hu, Liwei Lu, Meng Cao, Li-xin Wang

**Affiliations:** 1 Department of Microbiology and Immunology, Medical School of Southeast University, Nanjing, Jiangsu Province, People’s Republic of China; 2 Cancer Research and Biotherapy Center, the Second Affiliated Hospital of Southeast University, Nanjing, Jiangsu Province, People’s Republic of China; 3 Laboratory of Cancer Immunobiology, Earle A. Chiles Research Institute, Providence Portland Medical Center, Portland, Oregon, United States of America; 4 Department of Pathology and Center of Infection and Immunology, The University of Hong Kong, Hong Kong, Special Administrative Region, People’s Republic of China; University of California, Riverside, United States of America

## Abstract

Previously, we have documented that isolated autophagosomes from tumor cells could efficiently cross-prime tumor-reactive naïve T cells and mediate tumor regression in preclinical mouse models. However, the effect of tumor-derived autophagosomes, here we refer as to DRibbles, on B cells has not been studied so far. At present study, we found that DRibbles generated from a murine hepatoma cell line Hep1-6, induced B-cell activation after intravenous injection into mice. B-cell populations were significantly expanded and the production of Hep1-6 tumor-specific antibodies was successfully induced. Moreover, in vitro studies showed that DRibbles could induce more efficient B-cell proliferation and activation, antibody production, and cytokine secretion than whole tumor cell lysates. Notably, we found that B-cell activation required proteins but not DNA in the DRibbles. We further showed that B cells could capture DRibbles and present antigens in the DRibbles to directly induce T cell activation. Furthermore, we found that B-cell activation, antibody production, cytokine secretion and antigen cross-presentation were TLR2-MyD88 pathway dependent. Taken together, the present studies demonstrated that tumor-derived autophagosomes (DRibbles) efficiently induced B cells activation, antibody production, cytokine secretion and antigen cross-presentation mainly depending on their protein component via TLR2/MyD88 dependent manner.

## Introduction

Autophagy is a cellular process in which portions of the cytoplasm are sequestered by double membrane vesicles termed autophagosomes [1]. With induction of autophagy and inhibition of lysosomal/proteasome activity, a broad spectrum of cellular antigens, including long-lived proteins, short-lived proteins, and defective ribosomal products (DRiPs), is sequestered in autophagosomes. These autophagosome enriched with DRiPs-containing blebs are termed DRibbles [2]. Our previous studies have shown that DRibbles are efficient carriers of protein antigens from tumor cells and tumor associated antigens encapsulated in the DRibbles could be captured by dendritic cells (DCs) and cross-presented to T cells [2]–[5].

B cells can recognize and respond to both soluble and membrane-associated antigens via specific B cell receptor (BCR) [6], [7]. Recent studies show that B cells express most Toll like receptors (TLRs) and can respond to a variety of TLR ligands [8], [9]. Following these stimuli, B cells can proliferate and differentiate into antibody secreting cells, becoming more efficient antigen-presenting cells or cytokine producer cells [10]. Antibodies are the first line defense against infection and most vaccines work because they elicit a protective antibody response. Therefore, it is highly desirable for vaccine to be able to induce strong B cell and T cell mediated adaptive immune responses. In addition to their role in humoral immunity, B cells are important professional antigen presenting cells (pAPCs) and in certain circumstance they are very efficient pAPCs for antigen cross-presentation [11], [12]. For the novel vaccines based on tumor-derived DRibbles, there is no available information concerning their effect on B cell function.

In this study, we examined whether tumor-derived DRibbles could induce B-cell activation and proliferation and production of tumor-specific antibodies in vivo. If so, we also set out to determine the molecular pathways by which DRibbles induce B-cell activation. Finally, we investigated whether B cells could uptake and cross-present DRibbles antigens and serves as efficient antigen presenting cells for T cell activation.

## Results

### DRibbles Induced Tumor Specific Antibody Production in vivo

**Figure 1:**
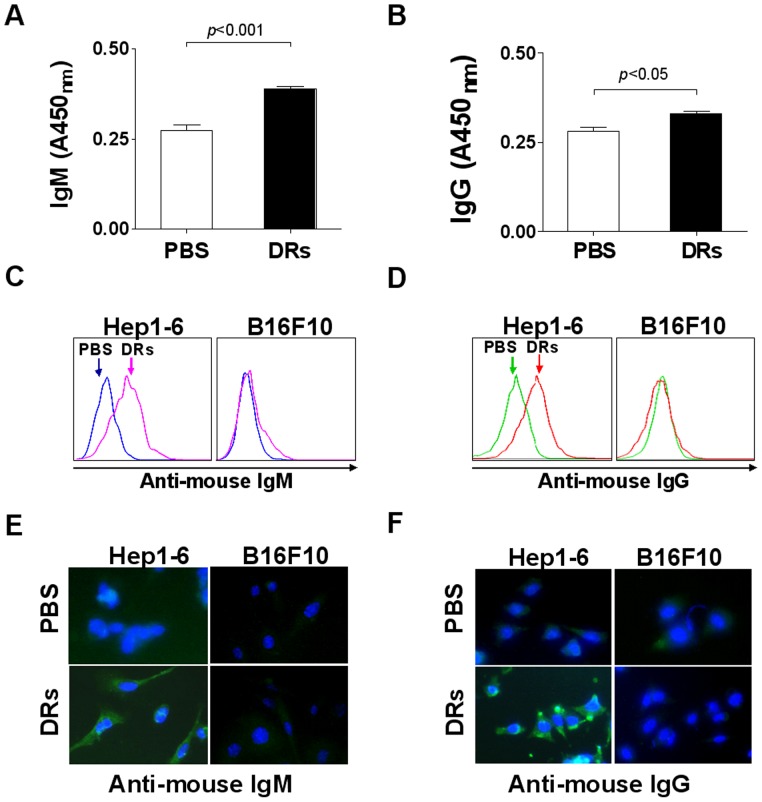
DRibbles derived from tumor cells induce specific antibody production in vivo. (A and B) Serum was collected from C57/BL6 mice at day 7 after first intravenous injection of PBS or Hep1-6 derived DRibbles (DRs). The total IgM (A) and IgG (B) in serum was measured by ELISA. Values are the mean ± SEM derived from three mice per group (n = 3). (C and D) Hep1-6 or B16F10 cells were incubated for 1 hour with serum derived from PBS or Hep1-6 DRibbles injected mice respectively. After washing, Hep1-6 or B16F10 cells were incubated with FITC-labeled anti-mouse IgM antibody (C) or FITC-labeled anti-mouse IgG antibody (D). After washing, the cells were analyzed by flow cytometry. (E and F) HepI-6 or B16F10 cells were treated as C and D, and then the cells were stained with DAPI and analyzed by fluorescence microscope. Green fluorescence represents FITC-labeled anti-mouse IgM antibody (E) or FITC-labeled anti-mouse IgG antibody (F). Blue fluorescence represents the cell nucleus. A representative of three independent experiments was showed.

To examine whether DRibbles could induce antibody production in vivo, C57/BL6 mice were injected intravenously with DRibbles derived from a murine hepatoma cell line (Hep 1-6) and then serum samples were collected at day 7 after first injection of DRibbles. ELISA analysis showed that levels of total serum IgM and IgG were significantly increased after injection with DRibbles comparing with PBS injection (**Figure 1A and B**). To further determine whether DRibbles-induced antibodies were specific to the antigens expressed by tumor cells, Hep1-6 or control cell line B16F10 cells were incubated with serum collected from Hep1-6/DRibbles-injected mice respectively, and then were stained with FITC-labeled anti-mouse IgM or IgG antibodies. Flow cytometric analysis showed that both IgM and IgG induced by Hep1-6 DRibbles were able to specifically stain Hep1-6 cells but not to B16F10 cells (**Figure 1C and D**). Consistently, immuno-ﬂuorescent microscopy also confirmed that IgM and IgG specifically stained to Hep1-6 cells, but not to B16F10 cells (**Figure 1E and F**). Subsequently, both reactivity and specificity of antibodies induced by Hep1-6/DRibbles were further detected by ELISA. It was found that the antibodies in the sera from Hep1-6 DRibbles-injected mice could react to Hep1-6 cells lysate, but not lysates of B16F10 cells or BNL cells compared with the control sera from PBS-injected mice (**Figure S1**). Thus, these results indicated that DRibbles derived from tumor cells could induce tumor antigen-specific IgM and IgG secretion in vivo.

### DRibbles Induced B cell Activation and Proliferation in vivo

**Figure 2:**
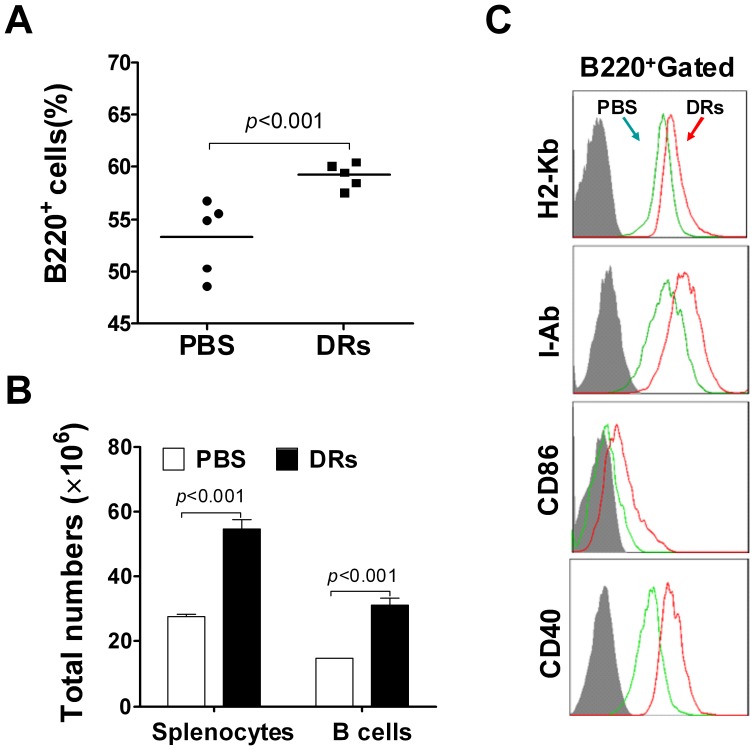
DRibbles induced proliferation and activation of B cells in vivo. (A and B) C57/BL6 mice were intravenously injected with DRibbles (DRs) or PBS as the control for 3 times in day 1, 2 and 3. Splenocytes were collected from each mouse in day 4 after first injection and were stained with anti-mouse B220 mAb. The percentage of B220^+^ cells in splenocytes (A) and total number of splenocytes and B220^+^ cells from each mouse (B) were analyzed by flow cytometry (n = 5). (C) Splenocytes from each mouse were stained with anti-mouse B220 mAb and the indicated mAbs respectively. The expression of H2-K^b^, I-A^b^, CD40 and CD86 of B220^+^ gated cells was analyzed by ﬂow cytometry. Gray shaded histograms indicate the isotype staining. Results represent three independent experiments.

To determine whether DRibbles-induced IgM and IgG production resulted from B cell activation, splenocytes were harvested from DRibble-injected mice, both frequencies and absolute numbers of B cells in the spleen were measured by flow cytometric analysis. Results showed that the percentage of B220^+^ B cells among total splenocytes was markedly increased after administration of DRibbles (**Figure 2A**). The total number of splenocytes or splenic B cells (B220^+^ cells) was significantly increased after injection of DRibbles comparing with PBS injection (**Figure 2B**). The activation status of B cells is commonly characterized by the expression of various activation markers, including the major histocompatibility complex (MHC), CD86 and CD40 [13], [14]. Flow cytometric analysis further revealed that the expression of MHC class I molecule (H-2K^b^), MHC class II molecule (I-A^b^), and co-stimulatory molecules (CD86 and CD40) on B cells was up-regulated after injection with DRibbles (**Figure 2C**). Taken together, these results indicated that DRibbles could induce B cells activation and proliferation in vivo.

### DRibbles Directly Induced B cell Proliferation and Activation in vitro

**Figure 3:**
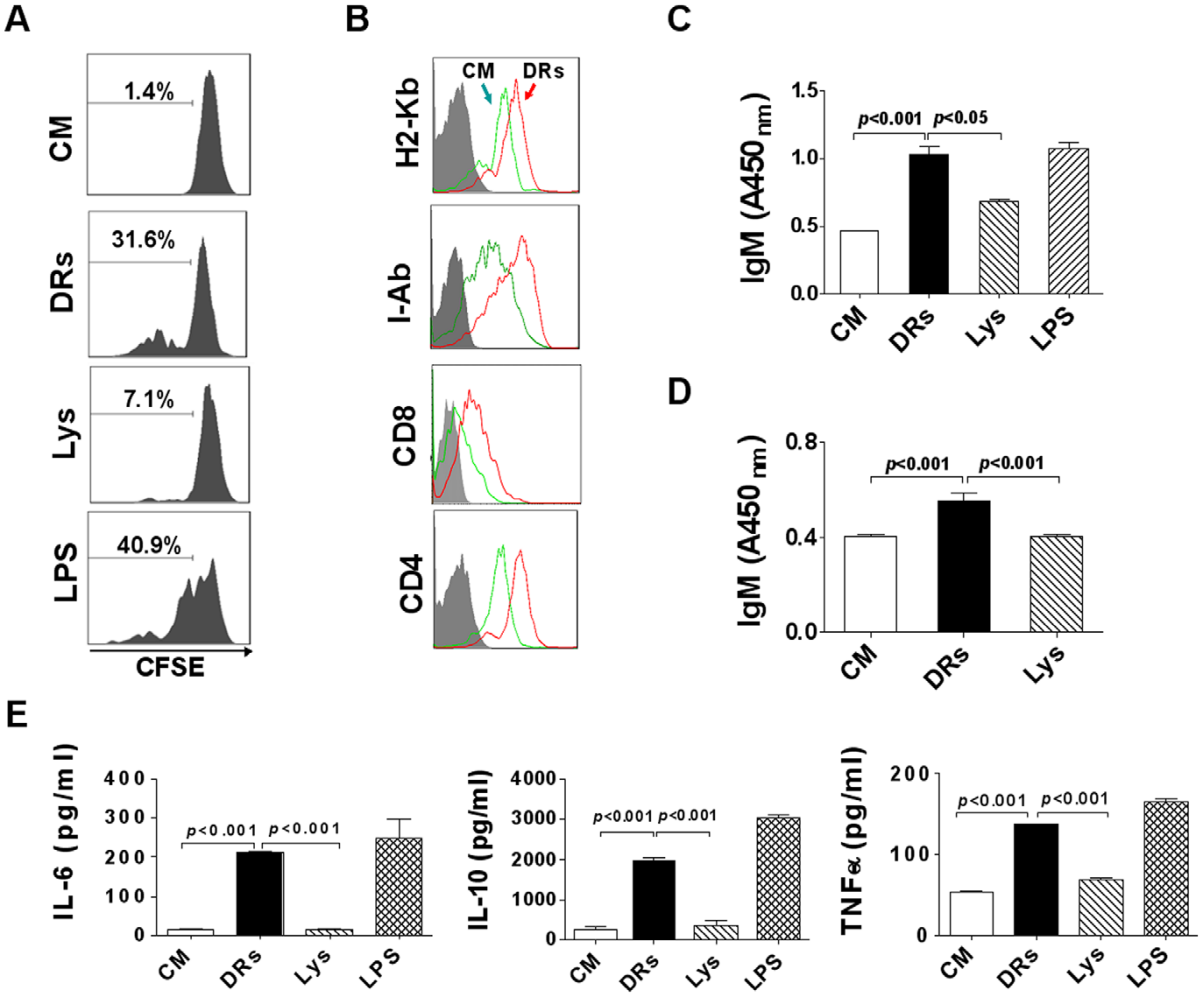
DRibbles induced proliferation and activation of B cells in vitro. (A) Purified B cells were labeled with CFSE and then co-incubated with DRibbles (DRs), whole tumor cell lysate (Lys) or LPS for 5 days. The proliferation of B cells was accessed by flow cytometry. (B) Purified B cells were co-incubated with DRibbles for 3 days and collected for staining with the indicated Abs or isotype-matched control Abs (gray filled area). The expression of H2-K^b^, I-A^b^, CD86 and CD40 on B cells was analyzed by ﬂow cytometry. (C and D) Splenocytes (C) and purified B cells (D) were co-incubated with DRibbles or lysate respectively for 7 days. IgM in the supernatants was analyzed by ELISA. (E) Purified B cells were co-incubated with DRibbles, tumor cell lysate or LPS for 3 days. Cytokines including IL-6, IL-10, TNF-αα in the supernatants was analyzed by ELISA. (CM indicated complete medium). Results represent three independent experiments.

B cell responses to most protein antigens require the activation of DCs and the recruitment of Ag-specific Th cells [15]. To further examine whether DRibbles or whole tumor cell lysate could stimulate B cell proliferation directly, B cells purified from naive mice were labeled with CFSE and incubated with DRibbles or tumor cell lysate for 5 days respectively. Flow cytometric analysis showed that DRibbles alone, similar to LPS, were higher potent at stimulating B cell proliferation than tumor lysate. Under our experimental condition, 31.6% of B cells were divided after stimulation with DRibbles and LPS caused 40.9% B cells to divide. In sharp contrast, tumor lysate at an equivalent protein concentration barely stimulated 7.1% B cells undergoing divisions (**Figure 3A**). To evaluate the direct effect of DRibbles on B cell activation, we cultured B cells in the presence of DRibbles for 72 hours and measured the expression of MHC molecules and co-stimulatory molecules on B cells by flow cytometric analysis. As expected, treatment with DRibbles significantly up-regulated the expression of MHC class I molecule (H-2K^b^), MHC class II molecule (I-A^b^), and co-stimulatory molecules (CD86 and CD40) on B cells (**Figure 3B**).

To determine whether DRibbles can induce antibody production by activated B cells in vitro, we cultured splenocytes and purified B cells in the presence of DRibbles or whole tumor cell lysate, and harvested the culture supernatants at day 7 for ELISA analysis of secreted antibodies. Results showed that stimulation of the splenocytes in vitro with DRibbles induced much higher levels of IgM secretion than with tumor lysate (**Figure 3C**). Moreover, in the absence of accessory cells, such as DCs and macrophages, the purified B cells could be stimulated by DRibbles to secrete significant higher levels of IgM secretion than that by tumor lysate (**Figure 3D**). Surprisingly, DRibbles did not increase the IgG level in the culture supernatants over that of control without stimulation (data not shown). These results showed that DRibbles, as a source of tumor antigens, were more efficient at activating B cells than whole tumor cell lysate.

Apart from antibody secretion, B cells are known to produce a wide range of cytokines, including IL-6, IL-10 and TNF-α [16], [17]. Next, we investigated whether DRibbles could also activate B cells to secrete pro-inflammatory cytokines. The purified B cells were cultured with DRibbles or whole tumor cell lysate for 72 hours, and the supernatants were harvested for cytokine detection. ELISA analysis showed that DRibbles stimulation, similar to LPS, could significantly increase the levels of IL-6, IL-10 and TNF-α, whereas whole tumor cell lysate stimulation could not (**Figure 3E**). However, very little IL-2 and IL-12, and no IFN-γ and IL-4 were detected when B cells were stimulated with DRibbles alone (**Figure S2**). These data demonstrated that DRibbles had a marked effect on the IL-6, IL-10 and TNF-α secretion of B cells.

### B cell Activation Requires Proteins, Not DNA, in DRibbles

**Figure 4:**
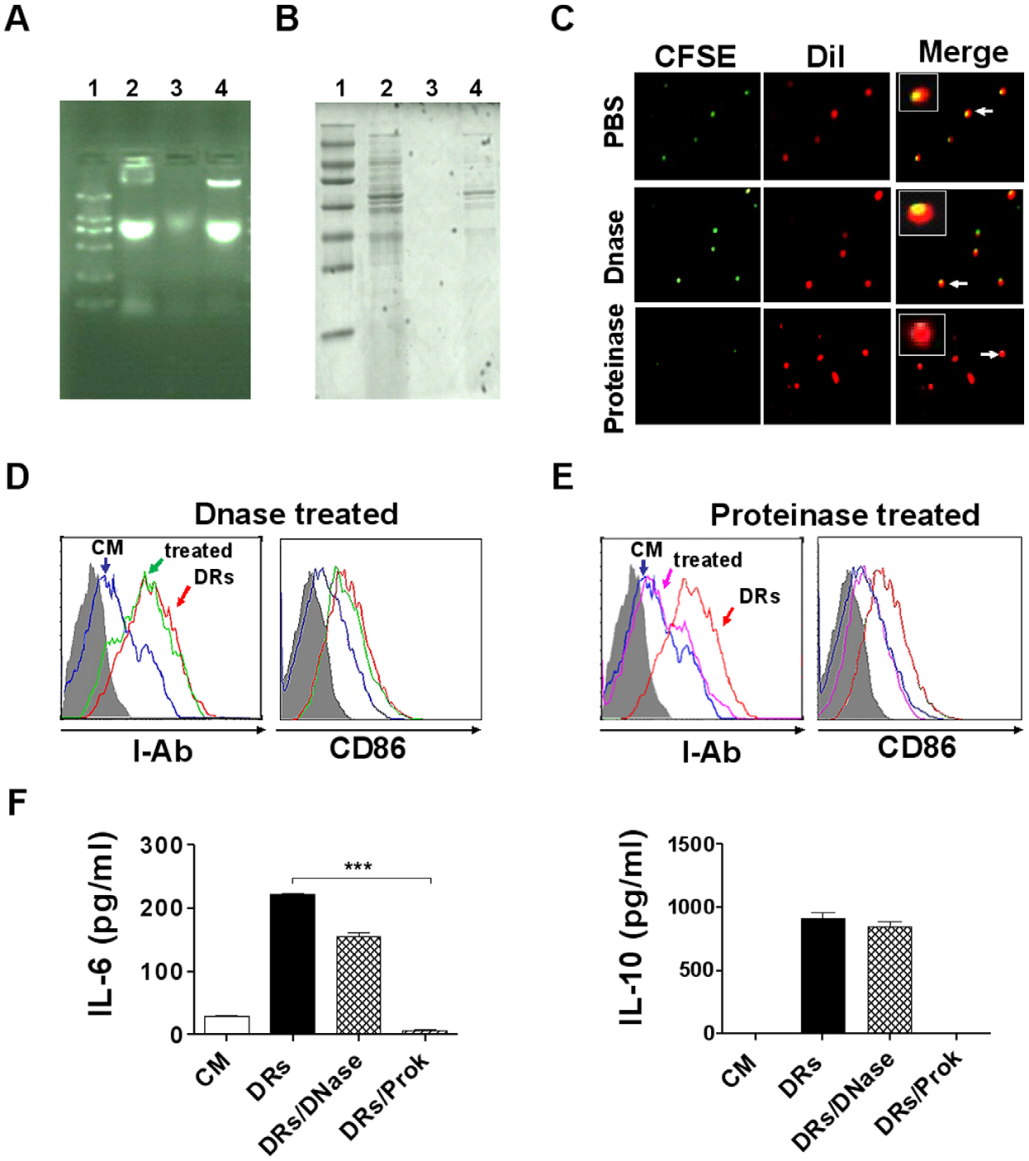
B cells activation was mainly depended on proteins but not DNA in DRibbles. DRibbles (DRs) were digested with DNase I or proteinase K and then released after lysed with RIPA lysis buffer. (A) The DNA in DRibbles was analyzed via agarose gel electrophoresis. (1. Markers 2. DRibbles 3. DRibbles digested with DNase I, 4. DRibbles digested with proteinase K). (B) The proteins in DRibbles were analyzed via 12% SDS polyacrylamide gel electrophoresis. (1. Markers 2. DRibbles 3. DRibbles digested with Proteinase K 4. DRibbles digested with DNase I). (C) DRibbles, DRibbles digested with DNase I or Proteinase K were stained with both CFSE and DiI. The characteristic of DRibbles was detected by the fluorescent microscope analysis (green presents CFSE labeled DRibbles-proteins; red presents DiI labeled DRibbles-membrane). (D and E) DRibbles digested with or without DNase I (D) or proteinase K (E) were co-incubated with purified B cells for 72 hours. B cells were collected and stained with Abs to corresponding surface markers (indicated), or isotype-matched control Ab (gray filled area). The expression of I-A^b^ and CD86 on B cells were analyzed by flow cytometry (blue line indicated complete medium; red line indicated DRibbles; green or pink line indicated DRibbles treated with DNase or proteinase). (F) B cells were incubated with DRibbles digested with or without DNase I or proteinase K for 72 hours, IL-6 and IL-10 in the supernatants were detected by ELISA. Results represent three independent experiments.

To test whether proteins or DNA contained in DRibbles cause B cell activation, we digested DRibbles with DNase I or proteinase K before they were used to stimulate B cells. Agarose gel electrophoresis showed that DNA in DRibbles was completely degraded upon digestion with DNase I (**Figure 4A**). Similarly, analyses by SDS-PAGE showed that proteins in DRibbles were completely degraded upon digestion with proteinase K (**Figure 4B**). Fluorescence microscopic examination showed that the membrane structure of DRibbles was not damaged when DRibbles was digested with DNase or Proteinase. CFSE-labeled DRibbles-proteins were completely eliminated by proteinase treatment, while Dil-labeled DRibbles-membrane remained intact. DNase treatment did not have any effect on either protein or membrane (**Figure 4C**). Subsequently, the purified B cells were incubated with digested or undigested DRibbles for 72 hours. Flow cytometric analysis showed that the expression of I-A^b^ and CD86 on B cells were still up-regulated when B cells were stimulated by DRibbles digested with DNase I, to a similar levels up-regulated by control DRibbles without digestion (**Figure 4D**), indicating that removal of DNA in DRibbles had no effect on B cell activation. By contrast, B cells failed to up-regulate the expression of I-A^b^ and CD86 when stimulated with proteinase-digested DRibbles (**Figure 4E**). In addition, ELISA analyses showed that there was no significant difference in the IL-6 and IL-10 secretion of B cells between undigested DRibbles and DNase-digested DRibbles stimulation. However, only small amounts of IL-6 and IL-10 were detected when B cells were stimulated with proteinase-digested DRibbles (**Figure 4F**). These results demonstrated that DRibbles-induced B cell activation mainly depended on the proteins, but not DNA component in DRibbles.

### DRibbles Induced B cell Activation via TLR2-MyD88 Dependent Manner

**Figure 5:**
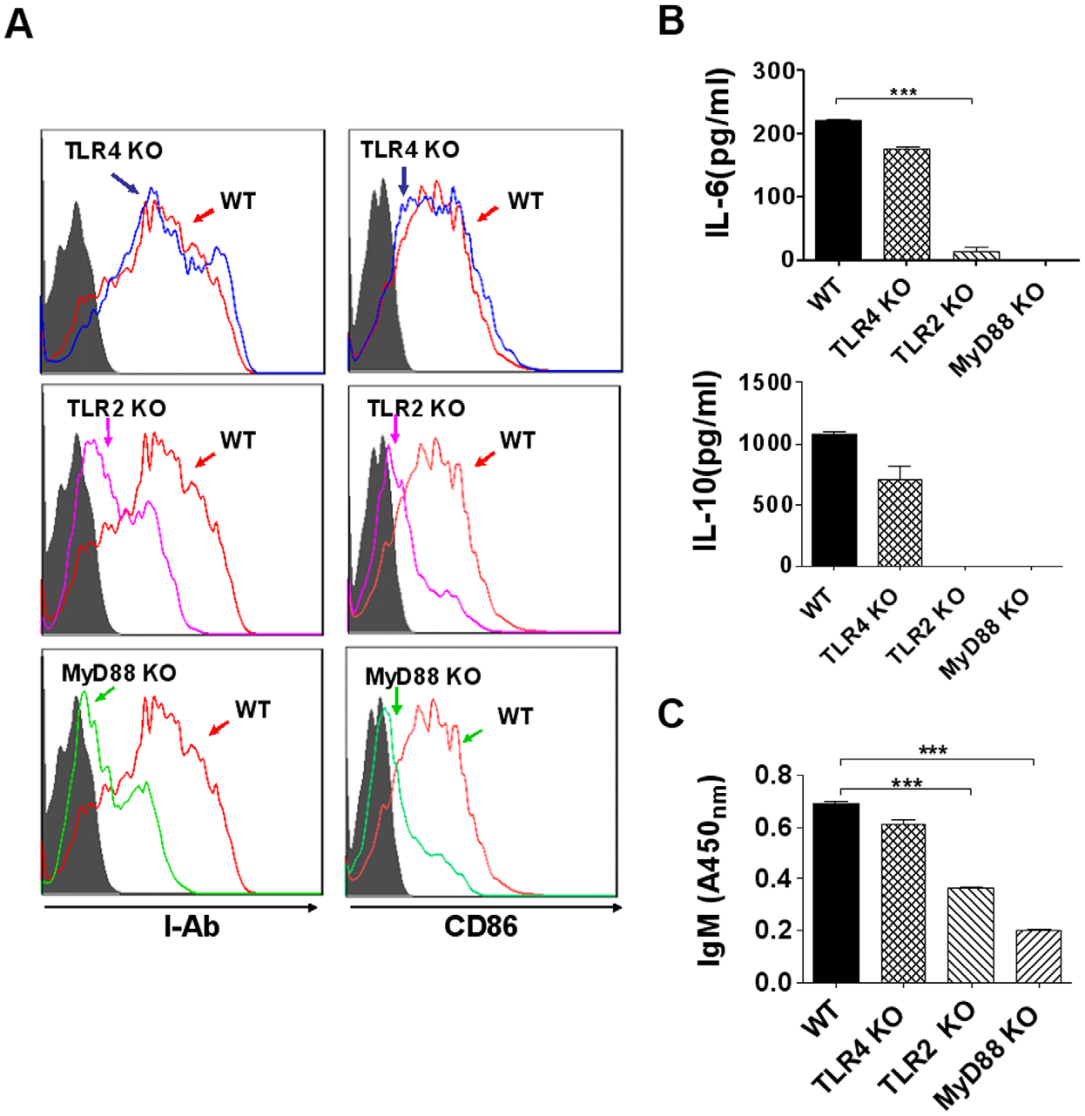
The role of TLR4, TLR2 and MyD88 signaling pathways in B cells activation induced by DRibbles. B cells purified from wild type, TLR4-, TLR2- or MyD88-deficient mice were co-incubated with DRibbles respectively for 72 hours. (A) B cells was collected and stained with indicated Abs. The expression of I-A^b^ and CD86 on B cells were detected by flow cytometer. (B) IL-6 and IL-10 in the supernatants were determined by ELISA. (C) IgM in the supernatants was determined by ELISA. (WT indicated B cells from wild type mice; TLR4KO, TLR2KO, and MyD88KO indicated B cells from TLR4-, TLR2- and MyD88-deficient mice). Results represent three independent experiments.

It has been shown that expression of MHC class II molecules and CD86 molecules on B cells was strongly up-regulated via TLRs stimulation [18]. In order to determine the role of TLR-mediated signaling pathways in B cell activation induced by DRibbles, B cells purified from wild type, TLR4-, TLR2- and MyD88-deficient mice were examined for MHC class II and CD86 expression after stimulation with DRibbles. We found that DRibbles failed to up-regulate I-A^b^ and CD86 expression on MyD88- or TLR2-deficient B cells, while TLR4 deficiency did not affect the up-regulation of I-A^b^ and CD86 expression on B cells (**Figure 5A**). Moreover, ELISA analyses showed that IL-6 and IL-10 secretion of B cells purified from TLR2- and MyD88-deficient mice was remarkably reduced compared with B cells from wild type mice. In contrast, TLR4-defficiency had no significant effect on IL-6 and IL-10 secretion when B cells were stimulated with DRibbles (**Figure 5B**). Interestingly, IgM production of B cells purified from TLR2- and MyD88-deficient mice, after stimulation with DRibbles, was markedly decreased when compared with that of TLR4−/− and normal B cells (**Figure 5C**). These results indicated that B-cell activation, antibody production, and cytokine secretion induced by DRibbles was TLR2-MyD88 dependent.

### B cells Could Directly Capture DRibbles in vitro

**Figure 6:**
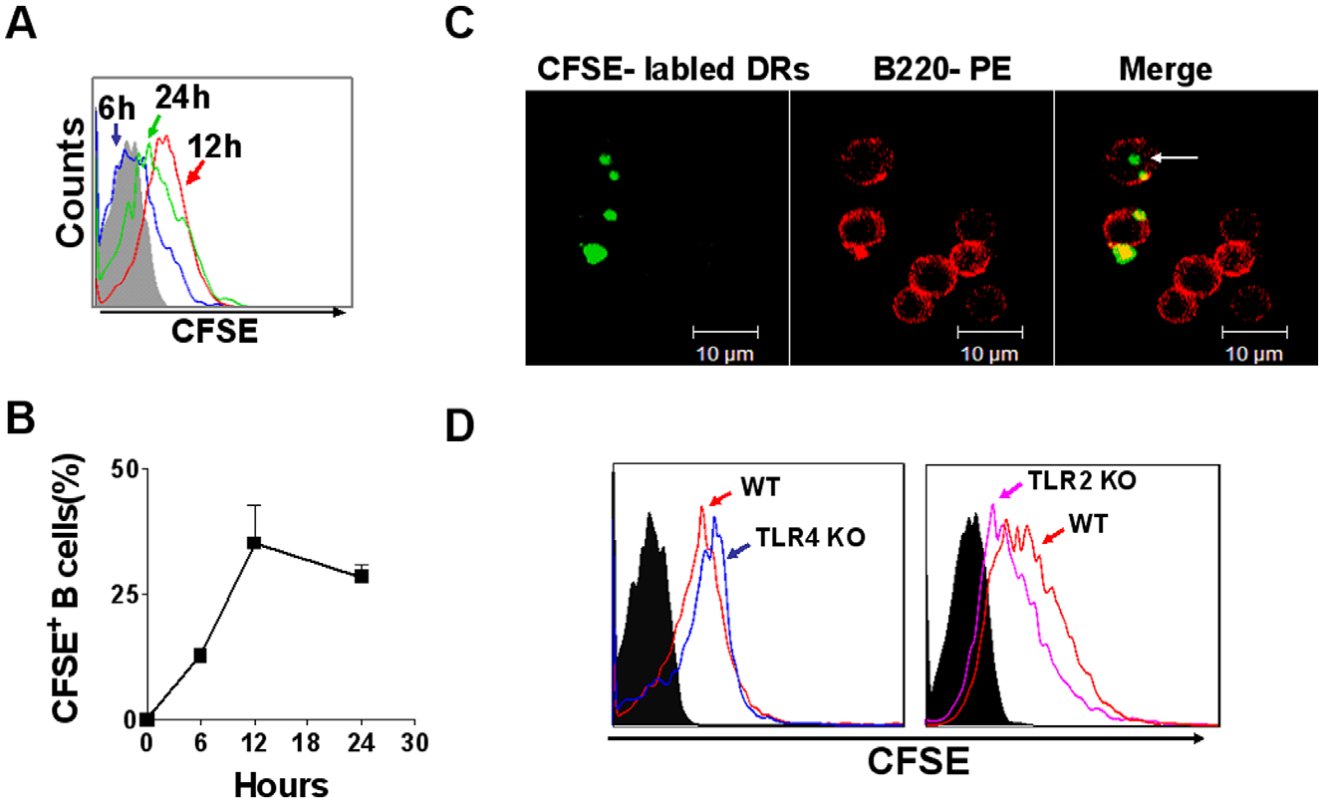
DRibbles can be taken up by B cells in vitro. (A) Purified B cells were co-incubated with CFSE-labeled DRibbles for 6, 12, 24 hours, CFSE positive B cells were assessed by flow cytometry. (B) The percentage of CFSE positive B cells were counted by flow cytometry as it has been indicated. (C) B cells taking up DRibbles were detected by the confocal laser microscope analysis. Representative images of immunofluorescence were stained with PE-conjugated anti-mouse B220 (red) and DRibbles labeled with CFSE (green). Arrows indicated that CFSE-labeled DRibbles were located inside of B cells. (D) B cells purified from wild type, TLR4- and TLR2-deficient mice were co-incubated with CFSE labeled-DRibbles for 12 hours, then the CFSE positive B cells were analyzed by flow cytometer. Data represent one of at least three experiments with similar results.

To investigate whether B cell could take up DRibbles directly, B cells were purified from the spleens of wild type mice and were incubated with CFSE-labeled DRibbles. Flow cytometric analysis showed that the frequency of CFSE-positive B cells was increased 6 hours after incubation and reached the peak level at 12 hours (**Figure 6A**). It is estimated that around 33% of B cells captured DRibbles after 12 hours of incubation. The frequency of CFSE-positive B cells decreased at 24 hours of incubation, possibly due to the degradation and disappearance of CFSE-labeled proteins (**Figure 6B**). Notably, confocal laser microscopy analysis revealed that CFSE-labeled DRibbles either attached to B cell membrane or entered into cytosol of B cells (**Figure 6C**). These results demonstrated that DRibbles could be efficiently captured and internalized by B cells.

To further determine the role of TLRs in the internalization of DRibbles, B cells purified from wild type, TLR4- and TLR2-deficient mice were incubated with CFSE-labeled DRibbles for 12 hours. Flow cytometric analyses showed that the capacity of TLR4^−/−^ B cells to capture DRibbles was similar to that of wild type B cells, but TLR2^−/−^ B cells showed a lower capturing capacity (**Figure 6D**). These results suggested that TLR2 might be involved in the DRibbles-uptake by B cells.

### B cells Could Present DRibbles-contained Antigens and Re-activated Effector T cells

**Figure 7:**
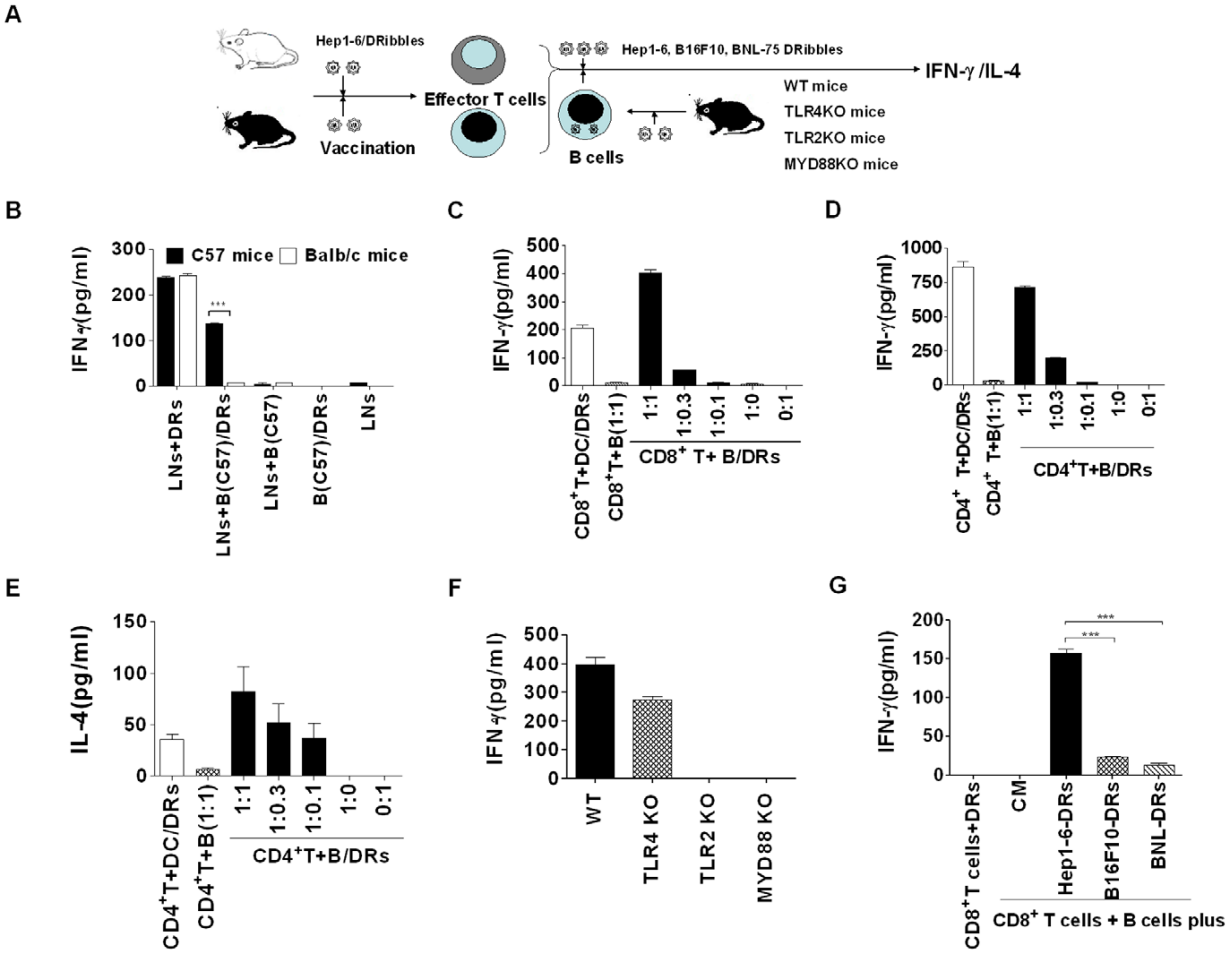
B cells loading DRibbles antigen could re-activate effector T cells. (A) The schematic diagram outlines the experiment protocol. C57/BL6 mice or BALB/c mice were intro-node vaccinated with DRibbles respectively. Lymphocytes were collected from lymph nodes (LN) of vaccinated C57/BL6 mice or BALB/c mice on day 7 after immunization. B cells purified from wide type C57/BL6 mice were stimulated with DRibbles for 6 hours and then washed 3 times with PBS. The lymphocytes were co-incubated with DRibbles or DRibbles-loaded B cells or B cells alone for 72 hours (n = 5). (B) The supernatants were harvested for detection of IFN-γ by ELISA. (C) CD8^+^ T cells were purified from the vaccinated C57/BL6 mice, and then co-incubated with B cells plus DRibbles (with DCs plus DRibbles at 1∶1 ratio or B cell alone as control) at the indicated ratio for 72 hours. IFN-γ in the supernatants was tested via ELISA. (D and E) CD4^+^ T cells were purified from the vaccinated C57/BL6 mice, and then co-incubated with B cells plus DRibbles at the indicated ratio for 72 hours. IFN-γ (D) and IL-4 (E) in the supernatants were tested via ELISA. (F) CD8^+^ T cells purified from the DRibbles-vaccinated C57/BL6 mice were co-incubated at 1∶1 ratio with B cells (from wild type, TLR4-, TLR2- or MyD88-deficient mice) plus DRibbles for 72 hours. IFN-γ in the supernatants was tested via ELISA. (G) CD8^+^ T cells were purified from the Hep1-6-DRibbles vaccinated C57/BL6 mice, and then co-incubated at 1∶1 ratio with B cells plus Hep1-6-, B16F10- or BNL-DRibbles for 72 hours respectively. IFN-γ in the supernatants was tested via ELISA. Data which are presented were obtained as a result of triplicates.

To examine whether B cells could process and present DRibbles antigens, we designed antigen cross-presentation experiment following the rules of MHC restriction, lymphocytes were harvested from DRibbles vaccinated C57/BL6 or BALB/c mice and then stimulated with B cells (from C57/BL6 mice) loading DRibbles (**Figure 7A**). IFN-γ in the supernatant was detected after 72 hours incubation. ELISA analysis showed that lymphocytes from both vaccinated C57/BL6 and BALB/c mice could secrete IFN-γ when stimulated with DRibbles directly. Interestingly, when C57/BL6 B cells were first loaded with DRibbles and washed away free DRibbles before they were used as APCs, only effector T cells from C57/BL6 mice but not BALB/c mice could respond and secrete IFN-γ (**Figure 7B**). These data showed that B cells could cross-present DRibbles-contained antigens to effector T cells independent of other accessory cells such as DCs or macrophages.

To determine the efficiency of cross-presentation of DRibbles-antigens by B cells, CD8^+^ T cells and CD4^+^ T cells were purified from the DRibbles vaccinated C57/BL6 mice and co-incubated with DRibbles-loaded DCs or B cells at the indicated ratios. ELISA analyses showed that DRibbles-loaded B cells induced IFN-γ secretion by CD8^+^ and CD4^+^ T cells in a B-cell number dependent fashion **(** **Figure 7C and D** **)**. However, only little IL-4 was detected when CD4^+^ T cells were co-incubated with B cells loading DRibbles (**Figure 7E**). When the same ratio (1∶1) was used, the cross-presentation efficiency between B cells and DCs appeared comparable. These results demonstrated that B cells could cross-present DRibbles -contained antigens and re-activate effector T cells response.

To further determine the role of TLRs in B cells in the cross-presentation of antigens in the DRibbles, B cells purified from wild type, TLR4-, TLR2- and MyD88-deficient mice were loaded with DRibbles and incubated with CD8^+^ T cells from the DRibbles-primed C57/BL6 mice. ELISA results showed that IFN-γ secretion of DRibbles-primed CD8^+^ T cells was greatly reduced when TLR2^−/−^ and MyD88^−/−^ B cells were used as APC as compared with WT B cells; whereas TLR4 deficiency in B cells had a weaker but significant effect on the IFN-γ secretion of CD8^+^ T cells (**Figure 7F**). These results suggested that the cross-presentation of antigens in the DRibbles by B cells was dependent on the TLR2-MyD88 pathway.

Finally, to determine whether the cross-presentation of antigens in the DRibbles by B cells was capable to stimulate antigen-specific T cells, DRibbles from Hep1-6, BNL cells, or B16F10 cells were loaded on B cells and used to re-stimulate Hep1-6/DRibbles primed CD8^+^ T cells. ELISA results showed that Hep1-6 DRibbles were efficiently cross-presented by B cells and induced IFN-γ secretion of Hep1-6/DRibbles-primed CD8^+^ T cells. However, only little IFN-γ release was detected when B cells were loaded with BNL cells- or B16F10 cells-derived DRibbles (**Figure 7G**). Our results suggested that DRibbles-loaded B cells could stimulate tumor -specific T cell immune response.

## Discussion

In this study, we have demonstrated that DRibbles could induce B cells activation and proliferation, which are consistent with the recognized function for DRibbles in activating antigen-specific T cells response [4], [5]. More importantly, DRibbles alone could increase IgM secretion in a T-independent manner in vitro. When antigens bear repeating determinants expressed at high density they can also activate specific B cells for further differentiation and antibody production in a T-independent manner [19]. DRibbles consisting of multiple epitopes or undefined antigens derived from tumor cells have given better clinical responses than single peptide or protein [20]. Similarly, the capacity of DRibbles to increase IgM secretion is more potent than whole cell lysate in vitro. T cell-dependent, BCR-driven responses lead to germinal center formation, affinity maturation, and memory cell differentiation, whereas TLR-driven responses tend toward rapid antibody-forming cell differentiation with limited affinity maturation and memory [21]. Here, we found that IgM production of B cells induced by DRibbles in vitro was dependent on TLR2-MyD88 signaling pathway. Moreover, there was no tumor antigen specific antibody production in vitro when purified B cells were stimulated with DRibbles (data not shown). However, the antibodies induced by DRibbles in vivo appeared to be tumor antigen specific. These results suggested that antibodies production induced by DRibbles in vitro depended mainly on TLR signaling pathway and these IgM might be nature antibodies that are not antigen specific, whereas the cooperation of BCR and TLRs may be required for antigen-specific antibody production induced in vivo.

B cells are major producers of cytokines such as IL-10, IL-6 and TNF-α [22]–[24]. Cytokines are a major component of cellular microenvironments, where they play a pivotal role in the control of cell survival, proliferation, and differentiation [25]. Our current results have revealed that DRibbles significantly enhance IL-10, IL-6 and TNF-α production by B cells. When stimulated with effector T cells and antigens, B cells have also been shown to produce detectable quantities of immune-regulatory cytokines such as IL-2, IL-12, IFN-γ and IL-4 [26]–[29]. In the current study, there is little IL-2 and IL-12 secretion when B cells are stimulated with DRibbles alone. However, no secretion of IFN-γ or IL-4 is detected when B cells are stimulated with DRibbles alone (data not shown). Recent studies show that IL-10-producing B cells possess a regulatory function both in vitro and in vivo [30]. Interestingly, we also found that DRibbles could induce IL-10^+^ B cells in vivo (data not shown). Further studies are needed to determine whether the cytokines produced by B cells regulate the activity or function of other cell types, especially T cells in tumor-bearing hosts.

DRibbles enrich a broad spectrum of tumor antigens, including intact protein, DRiPs and HSP-bound proteins [2]. Certain HSPs, such as HSP70, Gp96 played a critical role in preserving the antigen repertoire and up-regulating surface expression of MHC class II molecule and co-stimulatory molecules such as CD86. HSPs can activate DCs via TLR2/4 pathway [31]. Our data also demonstrated that the proteins in DRibbles played an important role in B cells activation. Recent studies showed that mouse B cells express TLR1–9, 11 and 13 [32]. TLRs ligation per se can provide the signals required for B cell proliferation, differentiation, and antibody secretion [21]. Most TLRs signal requires the adaptor protein MyD88, which initiate the classical NF-κB cascade [33], [34]. In this study, we found that MHC II and CD86 expression on B cells derived from MyD88 or TLR2 deficient mice were not up-regulated upon stimulation with DRibbles, while TLR4 deficiency barely affected B cells activation. Moreover, MyD88 or TLR2 deficiency severely affected the cytokine secretion and IgM production of B cells induced by DRibbles in vitro. Thus, our data have provided new evidence that DRibbles trigger B cell response via TLR2/MyD88 modulated manner.

Accumulated data show that B cells internalize antigens via at least two pathways: nonspecific fluid-phase pinocytosis and receptor-mediated endocytosis [35]–[37]. B cells take in the soluble fluid antigens via nonspecific fluid-phase pinocytosis. Our studies showed that splenic B cells could capture DRibbles directly. DRibbles are double-membrane bound vesicles with 300 nm to1 µm in size [4], it is unlikely that B cells capture DRibbles by pinocytosis. However, B cells are not considered as phagocytes, thus DRibbles uptake by B cells are also unlikely mediated by phagocytosis. Similar to other professional antigen-presenting cells, B cells express a variety of receptors like BCRs, complement receptors, FcγRs, and TLRs that are involved in the capturing of potentially antigenic materials [38]–[43]. Because B cells from TLR2-deficient mice take up fewer DRibbles than B cells from wild type mice, it is likely that B cells take up DRibbles through TLR2-dependent endocytosis pathway. Compared with TLR4, TLR2 is required for capturing of DRibbles by B cells and subsequent B cell activation. However, it is currently unknown whether other receptors such as BCRs, TLR7 and TLR9 affect internalization of DRibbles in B cells.

In addition to DCs or macrophages, B cells act as pAPC, can also take up and present T dependent antigens encapsulated in liposomes or associated with particles [44], [45]. When loaded onto DCs, DRibbles were efficient in activating naive CD8^+^ T cells, and DCs loaded DRibbles vaccine has potent antitumor efficacy [4]. Similarly, we found that B cells loaded DRibbles can cross-present DRibbles antigens and reactivate effector CD4^+^ and CD8^+^ T cells. B cells might process exogenous antigen via the class I pathway if antigens are taken up by receptor-mediated endocytosis [12]. Interestingly, our results showed that the cross-presentation of DRibbles-antigens by B cells was mainly dependent on TLR2-MyD88 pathway. Our results also provided strong evidence that B cells process and presented DRibbles-contained antigens via class I pathway, and predominately induced Th1 response. Previous studies have shown that DRibbles-loaded DCs vaccine could prime T cells and induce potent antitumor response [4], [5]. Our present results showed that Dribbles-loaded B cells also could induce T cells immune response, a process of being independent of DCs. However, the antitumor efficacy induced by B cells loaded DRibbles vaccine remains to be determined.

In conclusion, our studies have demonstrated that B cells can directly capture, process DRibbles and present DRibbles-contained antigen to T cells. DRibbles could induce B cell activation, antibody production, cytokine secretion in a TLR2/MyD88 modulated manner. Thus, further elucidation of the mechanism underlying DRibbles-induced B cell activation may facilitate the development of DRibbles vaccine to enhance antitumor efficacy in vivo.

## Materials and Methods

### Ethics Statement

All experimental protocols were approved by the Institutional Animal Care and Use Committee of Southeast University.

### Mice, Cell Lines and Reagents

C57BL/6 and BALB/c female mice were purchased from the Comparative Medicine Center, Yangzhou University (Yangzhou, China). TLR4-, TLR2- and MyD88-deficient mice were purchased from the National Resource Center for Mutant Mice Model Animal Research Center, Nanjing University (Nanjing, China). All mice were bred and maintained in specific pathogen-free conditions. Hep1-6 cells derived from the BW7756 hepatoma that arose in a C57L mouse were purchased from the Institute of Biochemistry and Cell Biology, Academy of Science (Shanghai, China). B16F10 melanoma cell line is syngeneic in C57BL/6 mice and was gifts from Dr. Dou Jun (Department of Microbiology and Immunology, Medical School of Southeast University, Nanjing, China) [46]. BNL cell line derived from BNL CL.2 (**TIB-75™**) by transformation with methylcholanthrene epoxide was purchased from ATCC. All the cells were cultured in complete medium made of RPMI 1640 (Gibico, USA) supplemented with 10% heat-inactivated FCS (Hyclone), 100 U/ml penicillin, 0.1 mg/ml streptomycin (Beyotime Institute of Biotechnology, Haimen, China). No mycoplasmas or filamentous fungu were detected in all reagents including DRibbles in the Clinical Laboratory of the Affiliated Zhongda Hospital of Southeast University.

### Isolation of DRibbles from Hep1-6 Cells

DRibbles were harvested from Hep1-6 cells as described previously [5]. Tumor cells were treated with 100 nM Rapamycin (Enzo Life Sciences, Shanghai, China), 100 nM Bortezomib (Millennium pharmaceuticals, Cambridge, USA), and 10 mM ammonium chloride in complete medium for 18–24 hours in a 5% CO2 incubator at 37°C. The cells were harvested and centrifuged at 1000 rpm. The supernatant was then centrifuged at 12,000 rpm to harvest the DRibbles. The total amount of protein in DRibbles was quantified by BCA protein assay Kit according to the manufacturer’s protocol (Beyotime Institute of Biotechnology, Haimen, China).

### Measurement of DRibbles Induced Tumor-specific Abs Production

Serum Abs generated in response to tumor cells was evaluated using an indirect immuno-ﬂuorescence assay and flow cytometry. C57/BL6 mice were injected intravenously (i.v.) with DRibbles (30 µg total protein) or PBS 3 times at day 1, 2 and 3. Sera were collected from the orbital sinus of mice at day 7 after first injection and stored at −20°C. B16/F10 cells and Hep1-6 cells were fixed in acetone at 4°C for 15 min, blocked in 1% BSA at 37°C for 1 hour, and then incubated with serum (1∶200) from DRibbles or PBS injected mice for 30 min at 4°C. The cells were then washed extensively, incubated with FITC-conjugated anti-mouse IgG or IgM Abs (Sigma Aldrich), and analyzed by ﬂow cytometry, or then the cells were subsequently stained with DAPI (Sigma Aldrich) and imaged under a ﬂuorescence microscope (Nikon-DXM1200, Nikon Instruments, Inc).

The reactivity of antibodies in the serum from Hep1-6-DRibbles-injected mice was measured by ELISA. Hep1-6, B16F10 and BNL tumor cell lysate were coated to plate at 10 mg/ml total proteins concentration overnight at 4°C. After washing with PBST (PBS plus 0.5% Tween-20), the plate were blocked with 5% goat serum in PBS for 2 h at 37°C. Then test serum was diluted serial dilutions and added to the plate for 1 h at 37°C. Subsequently, the antibodies were detected by incubation with HRP-conjugated anti-mouse IgM or IgG detection antibody (Jackson ImmunoResearch Laboratories), followed by TMB substrate solution for development of the ELISA. The optical density was measured at 450 nm.

B cells activation and proliferation in vivo were tested as following. C57/BL6 mice were injected intravenously with DRibbles or PBS as the control for 3 times at day 1, 2 and 3. Splenocytes were harvested from each mouse at day 4 after first injection and were stained with APC-labeled anti-mouse B220 mAb, PE-labeled anti-mouse H2-K^b^ (eBioscience), CD86, CD40 mAb and FITC-labeled anti-mouse I-A^b^ mAb (BD Bioscience). Purified anti-mouse CD16/CD32 Ab (BD Bioscience) was used to block non-specific binding to Fc receptors. Flow cytometric analysis was performed with an FACS Calibur (BD Bioscience), and the data was analyzed with Flowjo software (Tree Star). At least 10,000 live cell events gated by scatter plots were analyzed for each sample.

B cell proliferation in vitro was assessed by measuring the dilution of CFSE via flow cytometry. Purified B cells were isolated by negative selection using a combination of anti-CD43 coupled magnetic beads as proposed by the manufacturer (Invitrogen). In all cases, the purity of isolated B cells was ≥95% by flow cytometric analysis. Purified B cells from C57/BL6 mice were labeled with 5 µM of CFSE according to the manufacturer’s protocol (Invitrogen). CFSE-labeled B cells were co-incubated with DRibbles or whole tumor cell lysate (10 µg/ml total proteins) or LPS (10 µg/ml) for 5 days, and then single-cell suspensions were prepared and analyzed by flow cytometry (FACS Calibur, BD Bioscience).

Splenocytes were obtained from wild type C57BL/6 or TLR4-, TLR2- and MyD88-deficient mice. Single-cell suspensions were generated with cell viability higher than 98%. B cells were purified and stimulated with or without DRibbles (or whole tumor cell lysate, 10 µg/ml total proteins; or 10 µg/ml LPS). After 72 hours incubation, single cell suspensions were collected for detection of activation markers on B cell surface, and supernatants were harvested for detection of cytokines including IL-6, IL-10, TNF-α, IL-12, IL-2, IL-4 and IFN-γaccording to the ELISA manufactures protocol (eBioscience). After 7 days incubation, levels of IgM and IgG in the supernatants were measured by ELISA.

### IgM and IgG Detection by ELISA

ELISA was performed to detect mouse IgM and IgG in serum samples and cell culture supernatants according to the manufacturer’s protocol. Briefly, The plates were coated with either goat anti-mouse IgG or IgM (Jackson Immuno Research Laboratories) at 1∶10000 overnight at 4°C and washed with ELISA washing buffer (PBS plus 0.5% Tween-20). Then the plate were blocked with 2% goat serum in PBS and incubated with serial dilutions of test sera or cell cultured supernatants. Subsequently, total IgG or IgM was detected by incubation with HRP-conjugated anti-mouse IgG detection antibody or HRP-conjugated anti-mouse IgM detection antibody (Jackson ImmunoResearch Laboratories) respectively, followed by TMB substrate solution (Beyotime Institute of Biotechnology) for development of the ELISAs. Optical density was measured at 450 nm.

### Characterization of DRibbles Digested with DNase or Proteinase

DRibbles were digested with DNase I or proteinase K according to the manufacturer’s protocol (Beyotime Institute of Biotechnology), and then washed with PBS; complexes were released after lysed with RIPA lysis buffer (Beyotime Institute of Biotechnology). After lysing, treated or untreated DRibbles were centrifuged at 12,000 rpm, DNA or proteins in supernatant were analyzed via agarose gel electrophoresis or 12%SDS polyacrylamide gel electrophoresis.

After digestion with DNase I or proteinase K, DRibbles were stained with CFSE and DiI (Beyotime Institute of Biotechnology) according to the manufacturer’s protocol respectively. DRibbles were smeared on the glass slide, and then fixed in acetone at 4°C for 15 min; the characteristic of DRibbles was examined with a ﬂuorescent microscope (Nikon-DXM1200).

### Internalization and Cross-presentation of DRibbles Antigens by B cells

DRibbles were labeled with 5 µM CFSE according to the manufacturer’s protocol (Invitrogen), and then the purified B cells were co-incubated with CFSE-labeled DRibbles for 6, 12, 24 hours. Single B cells suspension were harvested and stained with PE-labeled anti-mouse B220 mAb (BD Bioscience), then were analyzed by flow cytometry and images were collected and analyzed on a Carl Zeiss confocal station using the LSM510 software (Carl Zeiss MicroImaging,Inc., Germany).

For DRibbles-contained antigens cross-presentation assay, C57/BL6 mice or BALB/c mice were vaccinated with DRibbles via intranodal. Lymphocytes were collected from lymph nodes of DRibbles vaccinated C57/BL6 mice or BALB/c mice, CD8^+^ T or CD4^+^ T cells were purified from above lymphocytes as effector T cells according to the manufacture’s protocols, respectively (Invitrogen). B cells were purified from wide type or TLR4, TLR2, MyD88 deficient C57/BL6 mice and stimulated with Hep1-6 DRibbles or BNL- or B16F10-DRibbles as control for 6 hours, washed 3 times with PBS. DCs were prepared by sequential intravenous injection of plasmid DNA encoding murine Flt3L and GM-CSF as described previously [4]. DRibbles loaded B cells or DCs were then co-incubated with CD4^+^T or CD8^+^T cells at the indicated ratio for 72 hours. IFN-γ or IL-4 in the supernatants was tested by ELISA according to the manufacturer’s protocol (eBioscience).

### Statistical Analysis

The mean ±S.E.M. was determined for each treatment group in the individual experiments. Two-tailed t-test was used to compare treatment groups with the control when significant differences were observed. Graphpad Prism 5.0 (Graphpad Software, San Diego, CA) was used for all statistical analysis.

## Supporting Information

Figure S1 The antibodies induced by Hep1-6-DRibbles in vivo reacted specifically with Hep1-6-DRibbles antigens. Hep1-6, B16F10 or BNL cell lysate were coated in plate, after blocking and washing, serum from PBS or Hep1-6-DRibbles injected mice was diluted 200 fold and added to the plate. Subsequently, IgM (A) and IgG (B) were detected by incubation with HRP-conjugated anti-mouse IgM or IgG detection antibody, followed by TMB substrate solution for development of the ELISA. Optical density was measured at 450 nm. Data represent at least three experiments with similar results.
(TIF)

Figure S2 DRibbles induced little IL-2 and IL-12 secretion of B cells in vitro. Purified B cells were co-incubated with DRibbles (DRs), tumor cell lysate (Lys) or LPS for 3 days. Cytokines including IL-2 (A) and IL-12 (B) in the supernatants was analyzed by ELISA. (CM indicated complete medium). Results represent three independent experiments.
(TIF)

## References

[1] Abeliovich H, Dunn WAJ, Kim J, Klionsky DJ (2000) Dissection of autophagosome biogenesis into distinct nucleation and expansion steps. J Cell Biol 151: 1025–1034. 11086004 10.1083/jcb.151.5.1025 PMC2174351

[2] Li YH, Wang LX, Pang P, Twitty C, Fox BA, et al (2009) Cross presentation of tumor associated antigens through tumor-derived autophagosomes. Autophagy 5: 576–577. 19333005 10.4161/auto.5.4.8366 PMC3128909

[3] Li Y, Wang LX, Yang G, Hao F, Urba WJ, et al (2008) Efficient Cross-presentation Depends on Autophagy in Tumor Cells. Cancer Res 68: 6889–6895. 18757401 10.1158/0008-5472.CAN-08-0161 PMC2905686

[4] Li YH, Wang LX, Pang P, Cui Z, Aung S, et al (2011) Tumor-derived autophagosome vaccine: mechanism of cross-presentation and therapeutic efficacy. Clin Cancer Res 17(00): 1–11. 10.1158/1078-0432.CCR-11-0951 PMC3495614 22068657

[5] Twitty CG, Jensen SM, Hu HM, Fox BA (2011) Tumor-derived autophagosome vaccine: induction of cross-protective immune responses against short-lived proteins through a p62-dependent mechanism. Clin Cancer Res 17: 6467–6481. 21810919 10.1158/1078-0432.CCR-11-0812 PMC3298078

[6] Carrasco YR, Batista FD (2006) B cell recognition of membrane-bound antigen: an exquisite way of sensing ligands. Curr Opin Immunol 18: 286–291. 16616474 10.1016/j.coi.2006.03.013

[7] Depoil D, Fleire S, Treanor BL, Weber M, Harwood NE, et al (2008) CD19 is essential for B cell activation by promoting B cell receptor-antigen microcluster formation in response to membrane-bound ligand. Nat Immunol 9: 63–72. 18059271 10.1038/ni1547

[8] Barr TA, Brown S, Ryan G, Zhao J, Gray D (2007) TLR-mediated stimulation of APCs: Distinct cytokine responses of B cells and dendritic cells. Eur J Immunol 37: 3040–3053. 17918201 10.1002/eji.200636483 PMC2699383

[9] Genestier L, Taillardet M, Mondiere P, Gheit H, Bella C, et al (2007) TLR agonists selectively promote terminal plasma cell differentiation of B cell subsets specialized in thymus-independent responses. J Immunol 178: 7779–7786. 17548615 10.4049/jimmunol.178.12.7779

[10] Gray D, Gray M, Barr T (2007) Innate responses of B cells. Eur J Immunol 37: 3304–3310. 18000957 10.1002/eji.200737728

[11] de Wit J, Souwer Y, Jorritsma T, Klaasse Bos H, ten Brinke A, et al (2010) Antigen-specific B cells reactivate an effective cytotoxic T cell response against phagocytosed Salmonella through cross-presentation. PLoS One 5: e13016. 20885961 10.1371/journal.pone.0013016 PMC2946406

[12] Ke Y, Kapp JA (1996) Exogenous antigens gain access to the major histocompatibility complex class I processing pathway in B cells by receptor-mediated uptake. J Exp Med 184: 1179–1184. 9064336 10.1084/jem.184.3.1179 PMC2192767

[13] Gantner F, Hermann P, Nakashima K, Matsukawa S, Sakai K, et al (2003) CD40-dependent and -independent activation of human tonsil B cells by CpG oligodeoxynucleotides. Eur J Immunol 33: 1576–1585. 12778475 10.1002/eji.200323444

[14] Lu H, Crawford RB, Kaplan BL, Kaminski NE (2011) 2,3,7,8-Tetrachlorodibenzo-p-dioxin -mediated disruption of the CD40 ligand-induced activation of primary human B cells. Toxicology and Applied Pharmacology 255: 251–260. 21807014 10.1016/j.taap.2011.06.026 PMC3189629

[15] Menard LC, Minns LA, Darche S, Mielcarz DW, Foureau DM, et al (2007) B cells amplify IFN-gamma production by T cells via a TNF-alpha-mediated mechanism. J Immunol 179: 4857–4866. 17878385 10.4049/jimmunol.179.7.4857

[16] Harris DP, Haynes L, Sayles PC, Duso DK, Eaton SM, et al (2000) Reciprocal regulation of polarized cytokine production by effector B and T cells. Nat Immunol 1: 475–482. 11101868 10.1038/82717

[17] Skok J, Poudrier J, Gray D (1999) Dendritic cell-derived IL-12 promotes B cell induction of Th2 differentiation: a feedback regulation of Th1 development. J Immunol 163: 4284–4291. 10510367

[18] Barr TA, Brown S, Ryan G, Zhao J, Gray D (2007) TLR-mediated stimulation of APC: Distinct cytokine responses of B cells and dendritic cells. Eur J Immunol 37: 3040–3053. 17918201 10.1002/eji.200636483 PMC2699383

[19] Serre K, Machy P, Grivel JC, Jolly G, Brun N, et al (1998) Efficient presentation of multivalent antigens targeted to various cell surface molecules of dendritic cells and surface Ig of antigen-specific B cells. J Immunol 161: 6059–6067. 9834089

[20] Neller MA, Lopez JA, Schmidt CW (2008) Antigens for cancer immunotherapy. Semin Immunol 20: 286–295. 18951039 10.1016/j.smim.2008.09.006

[21] Vos Q, Lees A, Wu ZQ, Snapper CM, Mond JJ (2000) B-cell activation by T-cell-independent type 2 antigens as an integral part of the humoral immune response to pathogenic microorganisms. Immunol Rev 176: 154–170. 11043775 10.1034/j.1600-065x.2000.00607.x

[22] O’Garra A, Stapleton G, Dhar V, Pearce M, Schumacher J, et al (1990) Production of cytokines by mouse B cells: B lymphomas and normal B cells produce interleukin 10. Int Immunol 2: 821–832. 1703785 10.1093/intimm/2.9.821

[23] Agrawal S, Gollapudi S, Su H, Gupta S (2011) Leptin activates human B cells to secrete TNF-α, IL-6, and IL-10 via JAK2/STAT3 and p38MAPK/ERK1/2 signaling pathway. J Clin Immunol 31: 472–478. 21243519 10.1007/s10875-010-9507-1 PMC3132280

[24] Pistoia V (1997) Production of cytokines by human B cells in health and disease. Immunol Today 18: 343–350. 9238838 10.1016/s0167-5699(97)01080-3

[25] Airoldi I, Guglielmino R, Ghiotto F, Corcione A, Facchetti P, et al (2001) Cytokine Gene Expression in Neoplastic B Cells from Human Mantle Cell, Follicular, and Marginal Zone Lymphomas and in Their Postulated Normal Counterparts. Cancer Res 61: 1285–1290. 11245421

[26] Kouskoff V, Famiglietti S, Lacaud G, Lang P, Rider JE, et al (1998) Antigens varying in affinity for the B cell receptor induce differential B lymphocyte responses. J Exp Med 188: 1453–1464. 9782122 10.1084/jem.188.8.1453 PMC2213405

[27] Kindler V, Matthes T, Jeannin P, Zubler RH (1995) Interleukin-2 secretion by human B lymphocytes occurs as a late event and requires additional stimulation after CD40 cross-linking. Eur J Immunol 25: 1239–1243. 7539752 10.1002/eji.1830250516

[28] Ohnishi E, Iwata T, Inouye S, Kurata T, Sairenji T (1997) Interleukin-4 production in Epstein-Barr Virus transformed B cell lines from peripheral mononuclear cells of patients with atopic dermatitis. J Interferon Cytokine Res 17: 597–602. 9355960 10.1089/jir.1997.17.597

[29] Sartori A, Ma X, Gri G, Showe L, Benjamin D, et al (1997) Interleukin-12: an immune-regulatory cytokine produced by B cells and antigen-presenting cells. Methods 11: 116–127. 8990097 10.1006/meth.1996.0395

[30] Yang M, Sun L, Wang S, Ko KH, Xu H, et al (2010) Novel function of B cell-activating factor in the induction of IL-10-producing regulatory B cells. J Immunol 184(7): 3321–3325. 20208006 10.4049/jimmunol.0902551

[31] Bolhassani A, Rafati S (2008) Heat-shock proteins as powerful weapons in vaccine development. Expert Rev Vaccines 7: 1185–1199. 18844593 10.1586/14760584.7.8.1185

[32] Tabeta K, Georgel P, Janssen E, Du X, Hoebe K, et al (2004) Toll-like receptors 9 and 3 as essential components of innate immune defense against mouse cytomegalovirus infection. Proc Natl Acad Sci USA 101: 3516–3521. 14993594 10.1073/pnas.0400525101 PMC373494

[33] Takeda K, Akira S (2004) TLR signaling pathways. Semin Immunol 16: 3–9. 14751757 10.1016/j.smim.2003.10.003

[34] Akira S, Takeda K (2004) Toll-like receptor signaling. Nat Rev Immunol 4: 499–511. 15229469 10.1038/nri1391

[35] Gosselin EJ, Tony HP, Parker DC (1988) Characterization of antigen processing and presentation by resting B lymphocytes. J lmmunol 140: 1408–1413. 3257975

[36] Singer DF, Linderman JJ (1990) The Relationship between Antigen Concentration, Antigen Internalization, and Antigenic Complexes: Modeling Insights into Antigen Processing and Presentation. J Cell Biol 111: 55–68. 2365735 10.1083/jcb.111.1.55 PMC2116156

[37] Wagle NM, Kim JH, Pierce SK (1999) CD19 regulates B cell antigen receptor-mediated MHC class II antigen processing. Vaccine 18: 376–386. 10506665 10.1016/s0264-410x(99)00207-8

[38] Zhang P, Li W, Wang Y, Hou L, Xing Y, et al (2007) Identification of CD36 as a new surface marker of marginal zone B cells by transcriptomic analysis. Mol Immunol 44: 332–337. 16616782 10.1016/j.molimm.2006.02.030

[39] Barnes N, Gavin AL, Tan PS, Mottram P, Koentgen F, et al (2002) FcgammaRI- deficient mice show multiple alterations to inﬂammatory and immune responses. Immunity 16: 379–389. 11911823 10.1016/s1074-7613(02)00287-x

[40] Crampton SP, Voynova E, Bolland S (2010) Innate pathways to B-cell activation and tolerance. Ann N Y Acad Sci 1183: 58–68. 20146708 10.1111/j.1749-6632.2009.05123.x PMC3422021

[41] Peng SL (2005) Signaling in B cells via Toll-like receptors. Curr Opin Immunol 17: 230–236. 15886111 10.1016/j.coi.2005.03.003

[42] Bekeredjian-Ding I, Jego G (2009) Toll-like receptors–sentries in the B-cell response. Immunology 128: 311–323. 20067531 10.1111/j.1365-2567.2009.03173.x PMC2770679

[43] Rozková D, Novotná L, Pytlík R, Hochová I, Kozák T, et al (2010) Toll-like receptors on B-CLL cells: expression and functional consequences of their stimulation. Int J Cancer 126: 1132–1143. 19685493 10.1002/ijc.24832

[44] Grivel JC, Crook K, Leserman L (1994) Endocytosis and presentation of liposome-associated antigens by B cells. Immunomethods 4: 223–238. 7820453 10.1006/immu.1994.1024

[45] Vidard L, Kovacsovics-Bankowski M, Kraeft SK, Chen LB, Benacerraf B, et al (1996) Analysis of MHC class II presentation of particulate antigens of B lymphocytes. J Immunol 156: 2809–2818. 8609400

[46] Zhao F, Dou J, He XF, Wang J, Chu L, et al (2010) Enhancing therapy of B16F10 melanoma efficacy through tumor vaccine expressing GPI-anchored IL-21 and secreting GM-CSF in mouse model. Vaccine. 28(16): 2846–2852. 10.1016/j.vaccine.2010.01.057 20153795

