# Supplementary figures and images for: Tumor-Derived Autophagosomes (DRibbles) Induce B Cell Activation in a TLR2-MyD88 Dependent Manner
Source: PLoS One. 2013 Jan 9;8(1):e53564. doi: 10.1371/journal.pone.0053564 (PMC3541185; doi:10.1371/journal.pone.0053564)

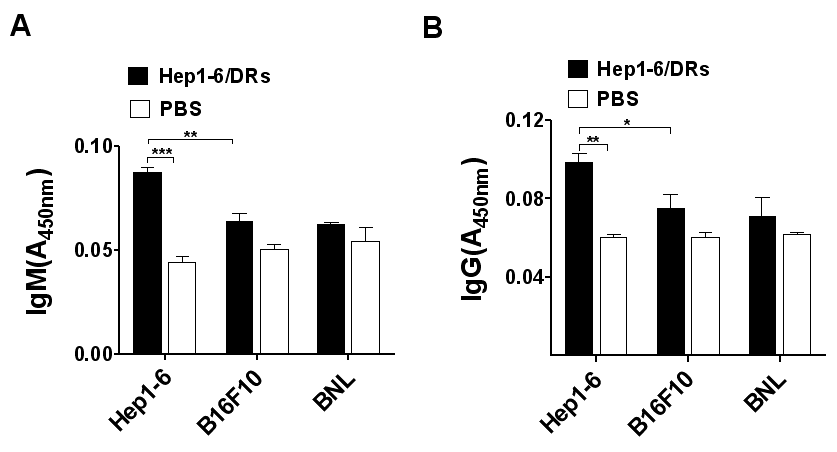

Supplement: Figure S1 — The antibodies induced by Hep1-6-DRibbles in vivo reacted specifically with Hep1-6-DRibbles antigens. Hep1-6, B16F10 or BNL cell lysate were coated in plate, after blocking and washing, serum from PBS or Hep1-6-DRibbles injected mice was diluted 200 fold and added to the plate. Subsequently, IgM (A) and IgG (B) were detected by incubation with HRP-conjugated anti-mouse IgM or IgG detection antibody, followed by TMB substrate solution for development of the ELISA. Optical density was measured at 450 nm. Data represent at least three experiments with similar results. (TIF) [file pone.0053564.s001.tif]

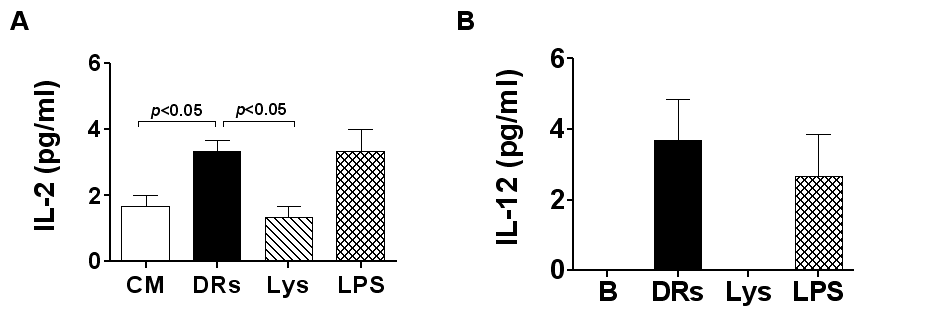

Supplement: Figure S2 — DRibbles induced little IL-2 and IL-12 secretion of B cells in vitro. Purified B cells were co-incubated with DRibbles (DRs), tumor cell lysate (Lys) or LPS for 3 days. Cytokines including IL-2 (A) and IL-12 (B) in the supernatants was analyzed by ELISA. (CM indicated complete medium). Results represent three independent experiments. (TIF) [file pone.0053564.s002.tif]
